# Metabolic and hormonal changes after laparoscopic sleeve gastrectomy in pediatric population: An observational study

**DOI:** 10.3389/fsurg.2022.1056458

**Published:** 2022-11-23

**Authors:** Hashim Alghamdi, Ashwag Asiri, Faris Alzahrani, Zainab Alamri, Yossef Hassan AbdelQadir, Jaffer Shah

**Affiliations:** ^1^ Mnistry of Health, Abha Maternity and Children Hospital, Abha, Saudi Arabia; ^2^ Department of Child Health, College of Medicine, King Khaled University, Abha, Saudi Arabia; ^3^ Research Center for Advanced Materials Science, King Khalid University, Abha, Saudi Arabia; ^4^ The Joint Program of Postgraduate Studies in Public Health and Preventive Medicine, Ministry of Health, Asir, Abha, Saudi Arabia; ^5^ Faculty of Medicine, Alexandria University, Alexandria, Egypt; ^6^ Kateb University, Medical Research Center, Kateb University, Kabul, Afghanistan

**Keywords:** obesity, endocrine, bariatric surgery, metabolism, thyroid

## Abstract

**Introduction:**

Despite the growing popularity of laparoscopic sleeve gastrectomy (SG) for managing severe obesity in children, adolescents, and adults, there is a paucity of studies reporting the effects of SG on metabolic and hormonal outcomes in pediatric populations.

**Methodology:**

In this single-centre, retrospective study, we assessed nutritional biomarkers (hemoglobin, ferritin, iron profile, Vitamin B12, Vitamin D, and calcium), glucose homeostasis indicators (C-peptide, HbA1C, and random blood glucose), blood lipids (triglycerides and cholesterol components), hormones involved in the hypothalamic-pituitary-adrenal axis (cortisol and adrenocorticotropic hormone), and thyroid hormones (T3, T4, thyroid-stimulating hormone, and parathyroid hormone) preoperatively and 12-month after SG in children aged 5–15 years.

**Results:**

This study included 64 adolescents (mean age = 11.2 ± 2.3 years) who underwent laparoscopic SG. Significant reduction in circulatory C-peptide (−62.1%; *p *= 0.005), HbA1C (−10.9%; *p *= 0.001), random blood glucose (−15.4%; *p *= 0.036), and triglycerides (−39.4%; *p *= 0.003) were observed postoperatively at 12 months compared to baseline. Although we did not observe any changes in cortisol levels, adrenocorticotropic hormone levels declined significantly by −40.9% postoperatively (*p *= 0.033). However, cholesterol components, thyroid hormones, and nutritional biomarkers remained unchanged from baseline.

**Conclusions:**

Consistent with prior literature, our study demonstrates improvement or resolution of diabetes and hypertriglyceridemia in the year following SG. However, given that blood cholesterol components, nutritional biomarkers, and thyroid profiles remained unchanged warrants long-term monitoring of nutritional, metabolic, and endocrine factors in adolescents undergoing laparoscopic SG. To the best of our knowledge, this is the first study reporting the effects of SG on thyroid and hypothalamic-pituitary-adrenal axis hormones in pediatric populations.

## Highlights

• T2DM is attenuated one year after sleeve gastrectomy in adolescents.
• Circulatory triglycerides decreased by 39.4% postoperatively at 12 months
• Adrenocorticotropic hormone declined by −40.9% postoperatively.
• Cholesterol fractions and nutritional biomarkers remained unchanged at follow-up.
• Also, thyroid hormones at 1-year follow-up were comparable to baseline.

## Introduction

Childhood obesity is associated with several complications such as type 2 diabetes mellitus (T2DM), hyperlipidemia, hypertension, fatty liver disease, psychosocial problems, and premature mortality (1). The worldwide prevalence of obesity increased by 10-fold between 1975 and 2016 among children and adolescents aged 5–19 (2), and in 2016, an estimated 340 million in this age group were overweight or obese globally (3).

Few pharmacological interventions are available for the treatment of childhood obesity. Orlistat is approved by US Food and Drug Administration (FDA) and is recommended by the National Institute for Health and Care Excellence (NICE) guidance for use in adolescents over 12 years of age (1). Similarly, the European Medicines Agency (EMA) has approved the use of liraglutide for obesity management in adolescents over 12 years (1). Children younger than 12 years are now approed to use setmelanotide, a melanocortin 4 receptor (MC4R) agonist, which the FDA and EMA have recently approved for weight management in children aged >6 years, but only if proopiomelanocortin, proprotein convertase subtilisin/kexin type 1, or leptin receptor deficiencies have been confirmed (1).

Consequently, clinical management of severe obesity for most children under 12 years is limited mainly to bariatric surgery, which is indicated for patients with a BMI of >40 kg/m^2^ or a BMI of >35 kg/m^2^ with significant comorbidities and unsuccessful weight loss with lifestyle modification approach attempts (1, 4). Several systematic reviews and meta-analyses have shown that bariatric surgery in the pediatric population leads to a significant reduction in BMI after 12 months: According to recent reports, A reduction range of −16.6 to −23.5 kg/m^2^ was observed with Roux-en-Y gastric bypass (RYGB), while −13.5 to −14.5 kg/m^2^ range was observed with sleeve gastrectomy (SG), and −8.5 to −10.5 kg/m^2^ with adjustable gastric banding (AGB) (5–7). Lower reductions in BMI have been observed with all three procedures at 24 and 36 months compared to 12 months post-surgery, indicating the regain of some body weight in the long term (7, 8). Moreover, bariatric surgery has been shown to improve health-related quality of life (HRQoL) with the resolution of comorbidities such as hypertension, sleep apnea, T2DM, and dyslipidemia (5, 6, 9), although the resolution of T2DM may be more pronounced than other comorbidities in the long term (8, 9).

The efficacy of bariatric surgery in producing meaningful weight loss, resolution of comorbidities, and improving HRQoL has also been demonstrated in the adult population (10, 11). Additionally, studies with adults show positive endocrine outcomes after bariatric surgery (12, 13). Among hormones related to body weight regulation and glucose homeostasis, favorable effects have been observed with ghrelin, leptin, glucagon-like peptide 1 (GLP-1), Peptide YY (PYY), glucagon, insulin, and adiponectin (12, 13). Furthermore, normalization of hormones involved in the somatotropic (growth hormone and Insulin-like growth factor (1) and gonadal (gonadotropin-releasing hormone, follicle-stimulating hormone, luteinizing hormone, testosterone, estradiol, sexual hormone-binding globulin, and prolactin) axes have been reported after bariatric surgery (12, 13). However, only a few studies report the effects on hormones in the hypothalamic-pituitary-adrenal (HPA) axis, such as corticotropin-releasing hormone, adrenocorticotropic hormone (ACTH), and cortisol, with inconsistent findings and unknown long-term impact (12). On the other hand, bariatric surgery is known to detrimentally impact bone health due to exacerbated hyperparathyroidism with concomitant hypocalcemia and hypovitaminosis D (12, 13).

Given the immense significance of the endocrine system in the pathogenesis of obesity or obesity-related disorders and the importance of restoration of endocrine function for favorable long-term patient outcomes, some researchers recommend obligatorily monitoring hormonal profile changes in patients after bariatric surgery (14). However, while hormonal changes after bariatric surgery in adults have been widely reported, such studies in adolescents and children are scarce. Therefore, this single-site retrospective study aims to evaluate hormonal and metabolic changes after laparoscopic SG in the pediatric population.

## Methodology

### Study participants and setting

Data from adolescent patients under 15 years old who underwent laparoscopic SG with our team at Abha Maternity and Children's Hospital, Al Mahalah, Abha, Saudi Arabia, was collected from March 2017 to April 2022. Abha Maternity and Children Hospital is the main referral hospital for children in the southern region of the Kingdom of Saudi Arabia.

The protocol of this study was approved by the Institutional Review Board of King Khaled University. Also, informed consent was obtained from families of the adolescents included before starting of data collection.

Included patients were indicated for surgery only after meeting the criteria for bariatric surgery which was described in many literature sources as the guidelines of the American Society for Metabolic and Bariatric Surgery (ASMBS) which recommend patients to be of Body mass index more than 40 kg/m^2^. Patients were also candidates for surgery if their BMI was equal to or more than 35 kg/m^2^ only if it was associated with other comorbidities such as obstructive sleep apnea, non-alcoholic hepatosteatosis to be eligable candidates for surgery (1, 15).

### Biochemical assessments

Biochemical assessments, including nutritional biomarkers, glucose homeostasis indicators, blood lipids, hormones involved in the HPA axis (Cortisol and ACTH), and thyroid hormones, were conducted preoperatively and at the 12-month follow-up as part of routine obesity evaluation.

The following serum parameters were measured at our hospital's central laboratory: Vitamin D, Calcium, Random Blood Sugar, HbA1C, C-Peptide, ACTH, Cortisol level, Lipid profile, Thyroid function test, Parathyroid hormone, Iron profile, ferritin, and Vitamin B12.

### Data analysis

IBM SPSS version 26 (SPSS, Inc. Chicago, IL) was used to code, collate, organize, and analyze data. All statistical analysis was done using two-tailed tests, and a *P*-value of <0.05 was considered statistically significant. Descriptive analysis based on frequency and percent distribution was done by gender. All other variables, including age, blood parameters, thyroid profile, lipid profile, and HbA1c, were displayed as mean with standard deviation and median. Also, the change in HbA1c after surgery was assessed by gender. All study parameters were compared before and after undergoing SG, with the percent change computed for each variable and negative sign was used to indicate the direction of change. Paired *t*-test was used to assess statistical significance.

## Results

Sixty-four children who underwent laparoscopic SG at our hospital were included in this study, of which 53.1% were females (Table 1). The age of the study population ranged from 5 to 14 years, with a mean age of 11.2 ± 2.3 years and only 10 children equal or below 9 years old at time of surgery (Table 1). The mean preoperative BMI for our cohort was 44.9 kg/m^2^. Patients with BMI below 39 kg/m^2^ represented 15% (*n* = 10) of our sample.

**Table 1:** Characteristics of children who underwent laparoscopic sleeve gastrectomy.

Variables	*n*	%
Gender		
Male	30	46.9%
Female	34	53.1%
Age, years		
Range	5–14	
Mean ± SD	11.2 ± 2.3	
Mean BMI	44.941	

### Nutritional biomarkers

While post-operative changes in hemoglobin (−4.7%) and ferritin (−25.5%) were numerically lower, and iron profile (15.7%) was numerically higher postoperatively, these changes were not statistically significant (Table 2). Similarly, circulatory levels of vitamin D (0.3%) and calcium (6.6%) were numerically but non-significantly higher after surgery compared to baseline (Table 2). We did not measure vitamin B12 levels pre-operatively, although the mean post-surgery levels (179 ± 160 pg/ml) which lies within the normal range.

**Table 2:** Blood biochemistry of study participants before and after laparoscopic sleeve gastrectomy.

Variables	Pre-operative			Post-operative			% Change	*p*
	Mean	SD	Median	Mean	SD	Median		
Nutritional Biomarkers								
Hemoglobin, g/dl	13.2	1.3	13.2	12.6	2.6	13.0	−4.7%	0.255
Iron profile	46.1	20.2	57.0	53.3	32.2	47.0	15.7%	0.475
Ferritin, ug/l	46.9	39.0	40.7	35.0	37.8	21.5	−25.5%	0.320
Vitamin B12, pg/mL	-	-	-	179	160	147	-	-
25-OH Vitamin D, ng/mL	13.6	6.3	13.6	13.6	5.5	13.5	0.3%	0.982
Ca, mg/dl	9.0	1.8	9.4	9.5	.5	9.6	6.6%	0.114
Glucose Homeostasis								
C-peptide, ng/mL	6.4	6.1	4.3	2.4	1.1	2.2	−62.1%	***0*** **.** ***005*** ^a^
Random blood glucose, mg/dl	102.9	34.0	92.7	87.1	17.3	91.0	−15.4%	***0*** **.** ***036*** ^a^
HbA1C (%)	6.0	0.8	5.7	5.4	0.4	5.4	−10.9%	***0*** **.** ***001*** ^a^
Lipid profile								
Triglyceride, mg/dl	112.1	59.7	97.0	67.9	34.7	59.3	−39.4%	***0*** **.** ***003*** ^a^
Cholesterol, mg/dl	157.9	40.8	156.8	165.4	32.0	157.0	4.8%	0.484
LDL, mg/dl	92.7	16.0	92.7	92.0	0.0	92.0	−0.7%	0.978
HDL, mg/dl	55.8	9.2	54.9	54.7	0.4	54.7	−1.9%	0.887
Hypothalamic-pituitary-adrenal axis								
ACTH, pg/mL	36.6	27.9	28.9	21.6	13.8	17.6	−40.9%	***0*** **.** ***033*** ^a^
Cortisol, μg/L	297.9	174.1	249.4	284.4	165.5	219.5	−4.5%	0.780
Thyroid profile								
TSH, µUI/mL	4.5	2.4	4.1	4.0	2.8	3.3	−11.2%	0.494
T3, nmol/L	6.9	1.3	6.8	6.7	2.9	6.0	−3.1%	0.768
T4, nmol/L	14.8	3.6	14.9	15.1	2.7	15.6	1.7%	0.791
PTH, pg/ml	65.9	19.4	64.3	78.3	44.3	65.2	18.8%	0.252

^a^ *p* < 0.05 (significant). % Change: (post value-pre value) *100/pre value.

### Glucose homeostasis

**Figure 1:**
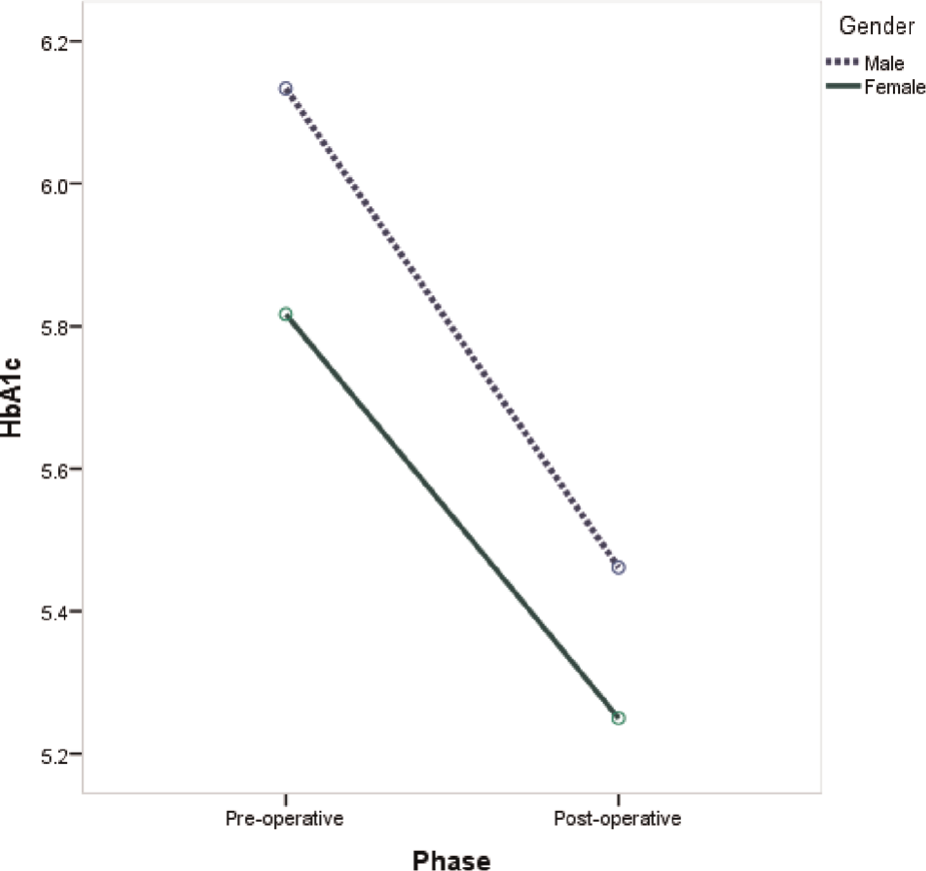
Change inn HbA1c before and after laparoscopic sleeve gastrectomy by gender.

Significantly lower levels of C-peptide (−62.1%; *P *= 0.005), HbA1C (−10.9%; *P *= 0.001), and random blood glucose (−15.4%; *P *= 0.036) were observed postoperatively at 12 months compared to baseline values (Table 2). Both male and female children showed a significant reduction in Hb1Ac levels after undergoing laparoscopic SG with the same reduction rate (−11% and −9.7%, respectively; *P *= 0.002) (Figure 1).

### Lipid homeostasis

Among blood lipids, serum triglyceride levels decreased significantly by −39.4% from 112.1 ± 59.7 mg/dl at baseline to 67.9 ± 34.7 mg/dl postoperatively (*P *= 0.003; Table 2). However, no statistically significant changes were observed in total cholesterol (4.8%), LDL-C (−0.7%), and HDL-C (−1.9%) levels postoperatively (Table 2).

### Hypothalamic-pituitary-adrenal axis

Among hormones involved in the HPA axis, we measured blood ACTH and cortisol (Table 2). A statistically significant reduction in ACTH levels of −40.9% was observed (from 36.6 ± 27.9 pg/ml at baseline to 21.6 ± 13.8 pg/ml postoperatively) (*P *= 0.033). Although post-surgery cortisol level was numerically lower (−4.5%), the difference wasn't statistically significant.

### Thyroid hormones

TSH (−11.2%) and T3 (−3.1%) levels were numerically lower while T4 (1.7%) and PTH (18.8%) were elevated 12 months post-surgery. However, none of these changes were of a statistical significance (Table 2).

## Discussion

In this retrospective study of children and adolescents who underwent laparoscopic SG, we observed significantly improved glycemic control, lower blood triglycerides, and lower circulating ACTH 12 months after surgery. However, other components of blood lipids such as LDL and HDL cholesterol, levels of thyroid hormones and factors related to bone metabolism, and nutritional biomarkers remained unchanged from baseline values.

Our findings, especially with metabolic outcomes, are consistent with earlier reports which investigated other surgeries such as RYGB and AGB, although studies specifically reporting the effects of SG are scarce. For instance, an earlier analysis of the Bariatric Outcomes Longitudinal Database (BOLD) showed that T2DM was attenuated in 78.6% of adolescents who underwent RYGB and in 59.1% of those who underwent AGB (16). However, resolution of hyperlipidemia was reported in only 58.8% and 23.3% of the adolescents with the two surgical procedures, respectively (15). Similarly, T2DM prevalence among Teen-LABS participants undergoing RYGB or SG decreased from 13.5% at baseline to 2.2% after one year, which was sustained for five years postoperatively (17). In contrast, total cholesterol decreased at one year but increased after that, while HDL-c increased at 1-year and 2-year follow-ups and was sustained at the 5-year follow-up (17). Furthermore, in the long-term Follow-up of Adolescent Bariatric Surgery-5+ (FABS-5+) study, 88% of adolescents who underwent RYGB had dyslipidemia compared to 27% prevalence after eight years with significant changes in median HDL-C (+62.6%), LDL-C (−12.3%), triglycerides (−25.5%) and TG/HDL-C ratio (−48.3%) (18).

We could identify only two prior studies that specifically report the resolution of comorbidities in children and adolescents after laparoscopic SG. Alqahtani et al. followed up SG recipients for three years and showed that 58.6% of comorbidities improved or resolved within three months post-surgery, which improved to 90.3% at the 2-year follow-up (19). During the 2- year follow-up, remission or improvement of LDL-C was noted in 78.1%, HDL-C in 82.7%, total cholesterol in 83.3%, triglycerides in 89.2%, hypertension in 86.3%, and T2DM in 100% of the participants (19). No further improvement in the rate of comorbidities resolution or the recurrence of comorbidities occurred in the third year after surgery (19). More recently, Elhag et al. reported the long-term cardiometabolic outcomes among adolescents after SG and impressively demonstrated remission of dyslipidemia, T2DM, and liver dysfunction in most patients within three years of surgery and for all patients at the 9-year post-procedural follow-up (20). To the best of our knowledge, ours is the first study to report the effect of SG on C-peptide and HbA1C, which expands the prior observations of the favorable effects of SG on glucose homeostasis. However, a significant decline in C-peptide and pro-insulin has been documented in adolescents 12 months after RYGB, along with improvements in insulin secretion, insulin sensitivity, glucose tolerance, and HbA1C (21, 22).

Further, no study to date has investigated the effects of SG (or any other bariatric procedure) on thyroid hormones or hormones involved in the HPA axis in adolescents. However, recent studies have demonstrated a positive association between fasting serum cortisol with fasting glucose and triglycerides irrespective of adolescents' age, gender, or degree of adiposity (23). Although we did not observe any post SG changes in cortisol levels, ACTH was significantly lower at 12 months. Also, we did not detect any statistically significant changes in levels of TSH, T3, T4, and PTH 12 months after surgery.

However, adolescents in previous studies undergoing SG report lower PTH and higher 25-hydroxy vitamin D levels than observed in our study (24, 25). For instance, Weiner et al. reported baseline PTH levels of 42.95 ± 29.37 pg/ml among adolescents with significant decline at 6 (−3.2%) and 12 months (−6.8%) post SG (24) compared to baseline PTH of 65.9 ± 19.4 pg/ml observed in our study cohort which remained unchanged at 12 months. Weiner et al. also reported a higher baseline 25-hydroxy vitamin D (19.86 ± 8.24 ng/ml) compared to our study (13.6 ± 6.3 ng/ml), although the levels remained unchanged at 12 months in both studies (25). These results are possibly confounded by the fact that patients received vitamin D3 and calcium supplementations, as is typical after bariatric surgery, to counter the detrimental impact on bone health. However, circulatory levels of calcium were comparable between the study by Weiner et al. and the current study, which remained unchanged at 12 months. Xanthakos et al. reported nutritional deficiencies among adolescents undergoing RYGB or SG more comprehensively (24). In adolescents undergoing SG, ferritin levels did not significantly change one year after surgery, as also observed in our study, but significantly declined at the 5-year follow-up (24). However, consistent with our observations Xanthakos et al. reported non-significant changes in 25-OH Vitamin D and PTH at 1- or 5-year follow-up (24). In contrast, vitamin B12 declined significantly at one year but was not significant at the 5-year follow-up (24).

### Strengths and limitations

Since this study was conducted in the main regional referral hospital for children, we had a high retention rate. Moreover, we used our hospital's central laboratory for blood biochemistry which adds to the clinical validity of our study. However, our study is limited by the small sample size and lack of a control group which was not feasible due to ethical issues with withholding treatment. Further, our panel of hormonal assessment was not exhaustive, and there is some evidence for changes in gonadal hormones (26) and adipocytokines (27–29) after bariatric surgery. We were also limited with nutritional assessment, especially for B group vitamins, vitamin E, copper, and zinc which would have improved the robustness of our findings. Finally, we could not account for the effect of diet and/or physical activity on our study variables.

## Conclusion

In conclusion, our study demonstrates improvement or resolution of T2DM and triglycerides among adolescents after 12 months of undergoing laparoscopic SG, consistent with prior literature. To the best of our knowledge, this is the first study reporting the effects of SG on thyroid hormones and hormones involved in the HPA axis. While ACTH was significantly lower, levels of cortisol and thyroid hormones did not change at the one-year follow-up. Given that blood cholesterol components and nutritional biomarkers remained unchanged in the year following surgery, long-term monitoring of nutritional, metabolic, and endocrine factors in adolescents undergoing laparoscopic SG is warranted.

## Data Availability

The original contributions presented in the study are included in the article/Supplementary Material, further inquiries can be directed to the corresponding author/s.
